# Implementation Adherence and Perspectives of the Childcare PhysicaL ActivitY (PLAY) Policy: A Process Evaluation

**DOI:** 10.1177/1090198121996285

**Published:** 2021-03-20

**Authors:** Monika Szpunar, Andrew M. Johnson, Molly Driediger, Shauna M. Burke, Jennifer D. Irwin, Jacob Shelley, Brian W. Timmons, Leigh M. Vanderloo, Patricia Tucker

**Affiliations:** 1 Western University, London, Ontario, Canada; 2 McMaster University, Hamilton, Ontario, Canada; 3 The Hospital for Sick Children, Toronto, Ontario, Canada

**Keywords:** childcare, early childhood educators, physical activity, policy, young children

## Abstract

The Childcare PhysicaL ActivitY (PLAY) policy was an evidence-informed, eight-item institutional-level policy document targeting children’s physical activity, outdoor play, and sedentary time. Nine childcare centers in London, Ontario, participated in this cluster, randomized controlled trial. Early Childhood Educators allocated to the experimental group, from five childcare centers in London, Ontario, implemented the policy for young children (18 months to 4 years) for 8 weeks and documented adherence to each policy item (i.e., dose) in daily logs. Program evaluation surveys (*n* = 21) and interviews (*n* = 10) were completed postintervention to assess Early Childhood Educators’ perspectives of feasibility, context, enjoyment, communication between researchers and childcare staff, and likelihood of future implementation. Descriptive statistics were calculated, and thematic analysis was conducted. Adherence to policy items ranged from 16.5% (for delivery of shorter, more frequent outdoor periods) to 85.9% (for delivery of unstructured/child-directed play). Participants reported effective communication between the research team and childcare centers (0 = *not at all effective* to 5 = *very effective; M* = 4.20; *SD* = 0.83) but noted that they were unlikely to continue the implementation of more frequent outdoor periods (0 = *not at all likely* to 5 = *extremely likely; M* = 2.19; *SD* = 1.21). Interview themes included weather as a prominent barrier and the use of verbal prompts as a solution for implementing the policy. As this was a small and short-term intervention, this pilot study offers important insight on larger scale policy interventions aimed at increasing physical activity and minimizing sedentary time among children enrolled in childcare.

Physical activity, particularly time spent in moderate-to-vigorous intensity physical activity (MVPA) among young children, plays an important role in supporting health and development (Carson et al., 2017; Poitras et al., 2016; Saunders et al., 2016). The *Canadian 24-Hour Movement Guidelines for the Early Years* (0–4 years; Canadian Society for Exercise Physiology [CSEP], 2017) provide daily recommendations for physical activity and sedentary time among this cohort. In detail, the guidelines recommend that toddlers and preschoolers engage in at least 180 minutes of total physical activity (TPA) per day and that preschoolers focus on achieving at least 60 minutes of MVPA. Furthermore, the guidelines suggest that children younger than 2 years receive no screen time, while children older than 2 years be restricted to 60 minutes per day. Finally, regardless of age, all prolonged sitting should be limited to no more than 60 minutes at a time (CSEP, 2017). A strong understanding of these new guidelines, and knowing how to implement them, is important for individuals who provide care for young children.

Early childhood educators (ECEs) are often responsible for planning the daily schedules of children enrolled in childcare (Hesketh et al., 2017). As of late, roughly two thirds of Canadian children aged 1 to 4 years are enrolled in childcare (Statistics Canada, 2019) and spend a large portion of their waking hours (~29 hours/week; Bushnik, 2006) in these settings. Given that significant autonomy is placed on childcare centers to offer daily physical activity programming, it is important that ECEs are well-positioned to facilitate, encourage, and help ensure that many young children are meeting the aforementioned movement guidelines. Researchers have reported low levels of physical activity among children in childcare (i.e., 1.5 min/h of MVPA; Vanderloo et al., 2014), and high levels of sedentary time (55.8 min/h; O’Brien et al., 2018). In addition, young children have been found to engage in large amounts of screen-viewing (De Decker et al., 2012), and this has been observed within childcare settings (Vanderloo et al., 2014). One systematic review reported that in more than half the studies (*n* = 17) included, preschoolers in childcare exceeded the recommended amount of screen-viewing allowance (60 minutes per day as referenced in CSEP, 2017; Vanderloo, 2014). These numbers warrant attention, as childcare represents a primary setting for physical activity opportunities for many children, due to inaccessibility or few energetic play opportunities at home (Copeland et al., 2016).

Research has suggested that the introduction of physical activity policies in childcare centers may be an effective strategy in the promotion of higher intensity activity among children (Ward et al., 2009). A small number of studies have noted increased rates of physical activity among children enrolled in centers with a policy in place (Bell et al., 2015; Bower et al., 2008; O’Neill et al., 2017; Stephens et al., 2014). For example, Stephens et al. (2014) found that the introduction of a physical activity–specific policy in childcare centers (*n* = 110) in New York was positively associated with children’s time spent in MVPA. Similar findings were found in South Carolina, where higher levels of activity were found among children enrolled in childcare centers (*n* = 34) adopting a physical activity policy compared with children in control centers without a policy in North Carolina (*n* = 30; O’Neill et al., 2017). Components of the policies implemented in New York and South Carolina varied; however, both policies incorporated daily time requirements for teacher-facilitated play and physical activity engagement (O’Neill et al., 2017; Stephens et al., 2014). Although studies like those transpiring in the United States represent a step in the right direction, the policy environment in childcare centers remains underdeveloped. In fact, no study to the best of our knowledge has examined the feasibility of implementing a physical activity and sedentary time policy in Canadian childcare settings.

A recent systematic review proposed that, to maximize effectiveness, policy interventions in childcare should focus on modifying the physical environments of the center (e.g., reducing playground density, providing portable play equipment), providing opportunities for children to participate in structured physical activity, and ensuring that childcare staff have adequate training and understand the importance of role modeling (Stacey et al., 2017). Therefore, it is important that the implementation of physical activity–targeted policies in childcare include a balanced combination of the abovementioned constituents. The evidence-based, stakeholder-informed Childcare PLAY Policy pilot study implemented in childcare settings was created to this end. It aimed to (a) increase physical activity, specifically time spent in MVPA; (b) increase outdoor play opportunities; and (c) decrease/interrupt extended periods of sedentary time (Tucker et al., 2019). In addition, the policy addresses the importance of young children’s physical literacy development, encourages participation in both unstructured and structured physical activity, and encourages limits on screen-based technology exposure.

By way of a process evaluation, the purpose of the present study was to assess the pilot implementation of the Childcare PLAY Policy in childcare centers. As the success of a childcare intervention may vary based on matters such as program design or the level of implementation from personnel responsible for delivering the intervention, this study was informed by the process evaluation framework proposed by Saunders et al. (2005) and followed the implementation evaluation conducted by Driediger et al. (2018). Specifically, the following factors were considered: the quality and extent of intervention implementation (i.e., adherence and dose delivered), ECEs’ perspectives on the policy (i.e., feasibility, enjoyment, communication, and future implementation), and contextual factors such as barriers/facilitators regarding the policy’s implementation (Saunders et al., 2005).

## Method

### Study Design and Procedures

The Childcare PLAY Policy process evaluation employed a pilot, single-blind, cluster randomized controlled trial. Childcare centers were selected from an online listing of 55 eligible childcare centers in London, Ontario, Canada, and randomized to control or experimental groups using block randomization. Centers allocated to the control group (*n* = 4) maintained their daily programming, and centers assigned to the experimental condition (*n* = 5) implemented the evidence-based Childcare PLAY policy for 8-weeks during the fall of 2018. The current study is part of the larger Childcare PLAY Policy study; a detailed methodological account is outlined elsewhere (Tucker et al., 2019). The Health Sciences Research Ethics Board at Western University approved all study procedures and associated documents (REB #111890). The Clinical Trials Registry was provided by the U.S. National Library of Medicine (NCT03695523).

### Participants

ECEs who spoke and read English and provided care to children (18 months–4 years) in toddler or preschool classrooms from participating centers were eligible to participate. For this process evaluation, only educators who worked in childcare centers assigned to the experimental condition (i.e., those who delivered the intervention) were included.

### Protocol for Childcare PLAY Policy Intervention

Once consent was received from childcare directors and educators, 30-minute policy-related training from the research assistant was provided to all ECEs in the experimental condition at the center. ECEs were provided the flexibility and autonomy to implement and schedule the policy items within their daily curriculum/programming as they saw fit.

### Evaluation Components

This process evaluation examined seven implementation constructs, namely, adherence, dose delivered, context, feasibility, effectiveness and enjoyment, communication, and future implementation. See Table 1 for the Childcare PLAY Policy evaluation outcome variables and corresponding data source and analysis.

**Table 1.** Process Evaluation Outcome Variables of the Childcare PLAY Policy Intervention.

Evaluation variable	Question	Tool or procedure (data collection)	Data analyses
Adherence	To what extent was the policy implemented as intended?	Implementation log	% of classrooms offering all eight policy components 3×/week for 8 weeks
Dose delivered	To what degree were policy items incorporated to the daily curriculum?	Implementation log	% of classrooms offering all components 3×/week for 8 weeks
Context	What are the barriers/enablers of implementation?	Telephone interview; program evaluation survey	Descriptive statistics; themes identified through thematic analysis
Feasibility	To what extent was the intervention easy and convenient to implement?	Telephone interview; program evaluation survey	Descriptive statistics; themes identified through inductive and deductive content analysis
Perceived effectiveness and enjoyment	To what extent was the policy intervention (1) effective at increasing children’s physical activity and (b) enjoyable for both children and ECEs?	Telephone interview; program evaluation survey	Descriptive statistics; themes identified through inductive and deductive content analysis
Communication	How effective was the communication?	Program evaluation survey	Descriptive statistics
Future implementation	Are there any suggestions for future policy modification? What is the likelihood of future policy implementation?	Telephone interview; program evaluation survey	Descriptive statistics; themes identified through inductive and deductive content analysis

*Note.* Process Evaluation Framework adopted from Saunders et al. (2005). ECE = early childhood educator.

### Tools

#### Demographic Questionnaire

Administered at baseline, this survey collected ECEs’ demographic information including age, gender, ethnicity, education, and income; years of experience working in childcare; their self-reported physical activity behaviors; and perceived ability to positively role model physical activity behaviors. Means and standard deviations were calculated to describe ECEs’ demographic information.

#### Daily Implementation Log

To assess adherence and dose delivered, ECEs were asked to complete a 17-item daily implementation log on Monday, Wednesday, and Friday for each participating class during the 8-week intervention period. Designed for the purpose of this study, the 17-item log assessed implementation adherence (“yes/no/partly”) to each of the policy items. In the case that educators were unable to adhere to a specific policy item, they were to indicate the reason(s) (e.g., weather, child to ECE ratios, limited space, behavioral issues, or other). To explore ECEs’ adherence to the policy, and dose delivered of specific policy items, frequencies and percentage scores were derived from the implementation log. Composite scores were calculated by grouping items from the daily implementation log together to assess overall adherence to the eight policy items. Each policy item (*n* = 8) has 1 to 4 indicators used to create the composite scores (*n* = 17). In addition, each item of the implementation log was analyzed individually to assess dose delivered. Both adherence and dose delivered were calculated using frequency and percentage scores. Overall implementation adherence and dose delivered of the policy was calculated by summing the number of days when policy components (and implementation log items) were offered as intended across the 8-week intervention period. A percentage score was calculated for each item of the policy on a weekly basis. An average across the sample was produced for the composite scores and for “yes” responses to the individual items of the daily implementation log.

#### Program Evaluation Questionnaire and Educator Interviews

This 41-item survey, also developed for this study, and administered at postintervention, prompted ECEs to rate their satisfaction with the policy components on a 5-point Likert-type scale. The survey is in three sections: feasibility (20 items; e.g., ease of implementation; 1 = *strongly disagree* to 5 = *strongly agree*), future implementation (17 items; e.g., likelihood that participants will continue implementing policy components; 1 = *not at all likely* to 5 = *extremely likely*), and communication (four items; e.g., between research team and childcare staff; 1 = *not at all effective* to 5 = *very effective*). Finally, this survey included nine open-ended questions that gathered participants’ general thoughts (e.g., enjoyment and effectiveness), barriers encountered, and solutions used by ECEs during the policy implementation period.

During the last week of data collection, ECEs were invited to participate in a one-on-one telephone interview. ECEs who expressed an interest in participation were contacted via email, to discuss consent and arrange a convenient interview time. The interviews were conducted by a trained research assistant who followed a semistructured interview guide. During interviews, ECEs were asked to describe the challenges they encountered (e.g., barriers) and the solutions they used to overcome these challenges, make their suggestions for improving the policy, and explain their general experience with implementing the policy. Interviews took place after childcare hours and were scheduled to last approximately 30 minutes. Saturation was reached after eight interviews, although two additional interviews were conducted to confirm findings. Interviews were audio recorded and transcribed verbatim.

All 41 items in the program evaluation survey were assessed by calculating means and standard deviations. Interview data were analyzed using QSR NVivo (version 12) via thematic analysis to identify common responses (Anderson, 2010). Credibility was achieved through member checking and was used during interviews to help improve the accuracy and trustworthiness of responses (Guba & Lincoln, 1989). The data collected from the open-ended questions in the program evaluation survey and telephone interviews were used to identify recurring themes of contextual factors influencing policy implementation (barriers and facilitators), and ECEs’ opinions of the overall feasibility, likelihood of future implementation, enjoyment, and appropriateness of the policy.

### Data Analysis

All statistical analyses were performed using the Statistical Package for the Social Sciences (SPSS) program (version 25).

## Results

ECEs (*n* = 49; *M_age_* = 34.73 ± 12.04 years) from nine childcare centers participated in the Childcare PLAY intervention. Experimental group ECEs (*n* = 25), from five childcare centers (13 classrooms), were female (100%), Caucasian (60%), had a college degree (76%), and provided care for preschool-aged children (52%). In comparison with the control group, experimental group ECEs were more ethnically diverse and had fewer years of childcare experience. See Table 2 for full participant demographics.

**Table 2.** Early Childhood Educators’ Demographic Information (*n* = 49).

Participant characteristics	Intervention		Control	
	*n*	%	*n*	%
Sex				
Male	—	—	1	4.2
Female	25	100	23	95.8
Ethnicity				
Caucasian	15	60	21	87.5
Arab	3	12		
Latin-American			1	4.2
Asian	3	12		
Other	3	12	1	4.2
Prefer not to answer	1	4	1	4.2
Employment status				
Full-time	25	100	24	100
Children’s age-group				
Toddler	12	48	10	41.7
Preschool	13	52	14	58.3
Years of work experience				
<5 years	10	40	5	20.8
5–9 years	5	20	7	29.2
10–14 years	5	20	3	12.5
15–19 years	1	4	2	8.3
20+ years	4	16	7	29.2
Level of education				
High school	2	8	—	—
College	19	76	21	87.5
University	4	16	3	12.5

*Note*. Information is reported for participants who completed the demographic survey. All values shown may not add up to 100% or *n* = 25 (Intervention) or *n* = 24 (Control) due to missing data.

### Adherence and Dose Delivered

Implementation adherence (e.g., composite scores) and dose delivered (e.g., daily implementation log items) are presented in Table 3. Composite scores ranged from 12% adherence toward implementing more frequent outdoor periods to 93% for appropriate modeling of screen-viewing behaviors. Dose delivered of individual implementation log items ranged from 17%, for implementing more frequent outdoor periods, to 86% for engaging children in unstructured or child-directed play. See Table 3 for a detailed exploration of composite scores (i.e., adherence to the eight policy items) and dose delivered (i.e., each item of the implementation log analyzed individually) during the 8-week policy implementation period.

**Table 3.** Intervention Group Early Childhood Educators’ Conformity to Childcare PLAY Policy Individual Components (*n* = 16) and Composite Scores (*n* = 8) Based on Classroom Adherence (*n* = 13).

Policy item	Adherence to Childcare Play Policy items (%)																								*M*
	Week 1			Week 2			Week 3			Week 4			Week 5			Week 6			Week 7			Week 8			
	Y	P	N	Y	P	N	Y	P	N	Y	P	N	Y	P	N	Y	P	N	Y	P	N	Y	P	N	
Children engaged in PA *frequently*.	85	13	3	71	29	0	71	29	0	73	22	5	77	23	0	73	27	0	74	20	6	65	35	0	74
Children achieved a *minimum of 40 minutes* of heart-pumping energetic play.	49	44	8	63	27	10	63	27	10	54	37	10	68	33	0	62	27	11	54	30	16	60	38	3	59
^±^Encourage children to engage in higher intensity energetic play often throughout the day with a goal of accumulating a minimum of 40 minutes each day.	46			63			54			49			62			60			51			51			55
Children engaged in *indoor* PA.	59	33	8	58	40	3	58	40	3	63	29	7	60	33	8	73	14	14	54	32	14	68	19	14	62
Children participated in *outdoor* PA.	94	6	0	81	0	20	81	0	20	80	10	10	98	0	3	81	11	8	78	14	8	81	8	11	84
^±^Expose children to a variety of indoor and outdoor PA, including teacher-facilitated play daily.	61			53			53			50			60			54			43			49			53
Children engaged in *unstructured* or child-directed PA.	97	3	0	83	10	7	83	10	7	83	12	5	85	15	0	94	6	0	81	14	5	81	19	0	86
Children engaged in *structured* or teacher-facilitated PA.	77	18	5	83	12	5	83	12	5	85	12	2	78	23	0	81	19	0	73	24	3	60	30	11	78
Teachers participated in PA alongside children.	77	23	0	76	22	2	76	22	2	76	17	7	74	26	0	78	16	5	70	24	5	58	33	8	73
Teachers provided verbal prompts.	97	3	0	76	20	5	76	20	5	78	15	7	92	5	3	89	11	0	89	5	5	84	16	0	85
^±^Unstructured (i.e., child-directed) free play is predominant during outdoor time. When activity levels decline, childcare practitioners encourage continued energetic play through structured activity, participation alongside children, and use of verbal prompts.	67			63			63			66			69			75			68			42			64
Children received a *minimum of 120 minutes (2 hours)* of outdoor time.	82	5	13	63	0	38	63	0	38	63	10	27	90	0	10	72	11	17	73	11	16	51	27	22	70
Children were offered indoor active play *instead* of outdoor time.	21	16	63	31	23	46	31	23	46	27	20	54	20	20	60	30	11	60	25	8	67	35	8	57	28
^±^Outdoor time is offered for a minimum of 120 minutes each day unless extreme weather occurs. When extreme weather occurs, the opportunity exists for active play indoors.	13			18			13			10			15			17			14			5			13
Shorter (15–30 minutes) outdoor periods were offered.	8	11	82	12	5	83	12	5	83	12	24	63	15	18	68	27	8	65	30	5	65	16	11	73	17
More frequent (more than two) outdoor periods were offered.	21	0	80	27	0	73	27	0	73	15	5	81	25	0	75	25	3	72	22	3	76	16	3	81	22
^±^Short, frequent outdoor sessions are most conducive to higher intensity PA among children; therefore, short bouts (e.g., 15–30 minutes) of outdoor time are recommended often (e.g., 3–4 times a day).	5			3			12			2			15			19			22			14			12
Children practiced fundamental movement skills.	82	15	3	73	20	7	73	20	7	73	17	10	95	3	3	87	14	0	87	5	8	78	16	5	81
^±^Encourage children to develop physical literacy by practicing fundamental movement skills often throughout the day (e.g., running, skipping, hopping, or jumping).	82			73			73			73			95			87			87			78			81
^†^Children were exposed to staff using screen-based technology.	21	3	77	15	2	83	15	2	83	15	0	85	15	0	85	16	0	83	16	0	84	11	3	87	16
^†^Children used screen-based technology.	8	0	92	10	0	90	10	0	90	7	0	93	8	0	92	81	0	92	5	3	92	8	3	89	17
^±^ ^†^The appropriate use of screen-based technology is role modeled by childcare practitioners by avoiding it when children are present. Screen-based technology is not offered to children younger than 2 years of age and is not recommended during childcare hours.	8			8			7			7			8			8			5			8			7
Staff intentionally interrupted children’s time spent being sedentary (e.g., sitting, screen use).	33	28	39	44	15	42	44	15	42	51	17	32	54	15	31	43	24	32	51	14	35	38	22	41	45
^±^Programming is designed to break up sustained sedentary time using indoor movement–based activities.	33			38			44			51			54			43			51			38			44

*Note*. ^†^Represents reverse scored items. ^±^Represents composite scores. % reported corresponds to “complete” adherence (2.0). PA = physical activity; Y = yes; P = partial; N = no. Scores were computed from complete data received from participating classrooms. Shading represents grouped components and composite scores that were computed together for analysis.

### Feasibility, Future Implementation, and Communication

Via the program evaluation survey, 21 ECEs (84%) from the experimental group reported on the feasibility of the policy, future implementation, and effective communication. Scores regarding feasibility (1 = *strongly disagree* to 5 = *strongly agree; M_range_* = 2.14 to 4.67; *includes reverse scored items*) and future implementation (1 = *not at all likely* to 5 = *extremely likely; M_range_* = 2.19 to 4.71) varied between items. Mean scores in the effective communication (1 = *not at all effective* to 5 = *very effective; M_range_* = 4.00 to 4.25) category suggest that ECEs believed that communication in the study was very effective for all five items. The screen time components of the policy (avoiding ECEs’ use of screen-based technology during childcare hours and avoiding children’s exposure to screen-based technology during childcare hours) showed high feasibility (*M* = 4.32, *SD* = 1.20; and *M* = 4.67, *SD* = 0.69) and likelihood of future implementation (*M* = 4.58, *SD* = 0.77; and *M* = 4.68, *SD* = 0.67), respectively. In contrast, likelihood to provide children with shorter, more frequent outdoor periods was scored much lower (*M* = 2.19, *SD* = 1.21) by ECEs compared with all other items in the future implementation category. ECEs strongly agreed that feasibility of frequent outdoor sessions was difficult (*M* = 4.00, *SD* = 1.41) to implement. Means and standard deviations for all 41 items in the survey are shown in Table 4. See Table 5 for prominent themes and sample quotes from participants’ responses on the program evaluation survey’s open-ended questions.

**Table 4.** Descriptive Statistics for Early Childhood Educators’ (*n* = 21) Responses to the Program Evaluation Survey.

Item	*M*	*SD*
Feasibility^a^		
When first approached to participate, I was very receptive to implementing the policy.	3.8	0.83
I felt adequately prepared to implement the policy.	3.8	0.83
The policy was easy to implement.	3.7	0.86
It was *not* easy to encourage children to engage in physical activity frequently throughout the day.	2.4^†^	0.93
It was easy to frequently encourage higher intensity play throughout the day.	3.6	1.0
It was easy to provide children with at least 40 minutes of higher intensity play each day.	3.9	1.1
It was *not* easy to expose children to a variety of indoor physical activities each day.	2.9^†^	1.2
It was easy to expose children to a variety of outdoor physical activities each day.	4.3	0.66
It was easy to provide unstructured or child-directed free play each day.	4.4	0.60
It was *not* easy to provide structured or teacher-facilitated play each day.	2.4^†^	1.4
It was easy to offer a minimum of 120 minutes of outdoor time each day.	4.2	0.62
It was easy to provide the opportunity for children to engage in active play indoors when outdoor play was not possible.	3.4	1.1
It was *not* easy to provide shorter, more frequent outdoor play sessions.	4.0^†^	1.4
It was easy to encourage continued energetic play through structured or teacher-led activities.	3.8	0.87
It was easy to encourage energetic play through teacher participation in physical activity.	4.0	0.86
It was *not* easy to encourage continued energetic play using verbal prompts.	2.1^†^	0.96
It was easy to support children’s development of physical literacy through encouragement of fundamental movement skills.	4.3	0.59
It was easy to avoid using my own screen-based technology when the children were present.	4.3	1.2
It was easy to avoid children’s exposure to screen-based technology during childcare hours.	4.7	0.69
It was *not* easy to break up children’s sedentary time by providing indoor active play opportunities.	3.3^†^	1.1
Future implementation (I plan to continue to . . . )^b^		
Encourage children to engage in physical activity frequently throughout the day.	4.3	0.78
Encourage children to engage in higher intensity energetic play often throughout the day.	3.9	0.99
Provide children with the opportunity to achieve a minimum of 40 minutes of higher intensity energetic play each day.	4.1	0.94
Expose children to a variety of indoor physical activities each day.	3.8	0.87
Expose children to a variety of outdoor physical activities each day.	4.6	0.59
Provide unstructured or child-directed free play each day.	4.7	0.48
Provide structured or teacher-facilitated active play each day.	4.3	0.58
Offer a minimum of 120 minutes of outdoor time each day.	4.7	0.46
Provide the opportunity for children to engage in active play indoors when outdoor play is not possible.	3.9	1.1
Provide shorter, more frequent outdoor sessions.	2.2	1.2
Encourage continued energetic play through structured or teacher-led activities.	3.9	0.92
Encourage continued energetic play through teacher participation in physical activity.	4.2	0.87
Encourage continued energetic play through verbal prompts.	4.4	0.80
Support children’s development of physical literacy through the encouragement of fundamental movement skills.	4.2	0.83
Avoid my own use of screen-based technology when children are present.	4.6	0.77
Avoid children’s exposure to screen-based technology during childcare hours.	4.7	0.67
Break up children’s sedentary time by providing indoor active play opportunities.	3.6	1.1
Communication and timing^c^		
How effective was the communication between the research team and your center?	4.2	0.83
How effective was the communication between your director and the staff?	4.0	1.1
How effective was the communication between and among staff members?	4.2	0.72
How effective was the communication between staff and/or the director and parents?	4.1	0.85

*Note*. Mean scored from 1 to 5; *SD* = standard deviation. Respondents were asked to rate the above statements from ^a^1 (*strongly disagree*) to 5 (*strongly agree*); ^b^1 (*not at all likely*) to 5 (*extremely likely*); and ^c^1 (*not at all effective*) to 5 (*extremely effective*). ^†^Represents reverse scored statements. All values shown may not add up to 100% or *n* = 21 as some individuals chose not to answer certain questions.

**Table 5.** ECEs’ Perspectives on Challenges, Solutions, Feasibility, Intervention Effectiveness, Enjoyment, and Suggestions for Childcare PLAY Policy Implementation Improvement.

Question	Theme	Example quotes	
		Program evaluation survey	Interview
Challenges	Transitions	• “The PLAY policy was asking for too many transitions.”	• “To get them dressed, undressed, come up the stairs, in and out, they (children) wouldn’t understand.”
	Weather	• “When there was snow on the ground, it was harder for the toddlers to do physical activity in their snow suits and the ground was slippery”	• “I think the short outdoor sessions would be easier in warm weather like now when we don’t have to put on snowsuits and boots.”
	Behavioral issues	• “We have some very emotional children in our care that like that close contact with their providers.”	• “Sometimes all those transitions would be hard, but it just depends on the day and the children’s attitude.”
	Other programming	• “Sometimes the children take time to develop other skills needed for growth.”	• “It’s our ministry, we have so many other things that we have to do as well.”
	Childcare environment	• “Difficult when sharing spaces to accommodate.”	• “It’s not that we don’t want them to be running, it’s just the space wise it is hard.”
	Lack of ECE training	• “It would have been nice to have similar training like the SPACE study”	• “I have a lot of experience and training. . . . I don’t see much of an issue keeping them active . . . but I know for other ECEs it could be.”
Solutions	Indoor PA	• “I found that on days where weather was bad, and I would take the children inside to split up the time.”	• “We’re going to the gym. We’re doing things in the hallways. So, instead of going outside, we were doing something inside.”
	ECE role modeling/encouragement	• “In my opinion, it took a lot of encouragement to get them active and physical.”	• “Because when they see you do things, they like to do them too, they like to be involved.”
	Structured PA	• “Small groups allow for more child and provider lead activities”	• “When we were setting up activities, then they were more inclined to do something active.”
Feasibility	More frequent outdoor sessions (15–30 minutes)	• “I don’t know how it would be possible to do short, frequent outdoor sessions. It takes 30 minutes to get toddlers ready for outside in the winter.”	• “If we were to take them out in 30-minute intervals, it would disrupt their play time.” “To get outside in 30-minute increments . . . I don’t think we have as much time as you think we do.”
	Outdoor PA	• In the winter the outdoor time got reduced to 80 minutes and less from 120 minutes. As the children took more time to get ready (because of snowsuits).	• “I think that part of it was kind of a little bit easier for us because we have a yard that’s kind of nice. It’s these other centers that sometimes don’t have those kinds of structures out there, right?”
	Screen time	• “The children who attend our center do not have any exposure to screen-based technology of any kind.”	• “We don’t have screen-based technology at all here, so that was easy.”
	MVPA	• “It was not easy to implement 40 minutes energetic play as my group is too young (toddlers).”	• “It is hard to get them to move vigorously because their attention span is very short.”
Perceived effectiveness	Childcare PLAY intervention	• “The policy made me see we put more value on brain activity over physical activity. This needs to change.”	• “Pushed me to encourage activeness of children.”
Enjoyment	Participants	• “Active toddlers make for better sleepers.” • “Children were happy and active when they engaged in physical activity.”	• “The parents were actually asking those questions. Like “What have you been doing? Like “Why is this working?” and “Are you noticing a difference because we’re noticing a difference at home?”
Suggestions for improvement	# of outdoor periods	• “Not do shorter outdoor times especially in winter.”	• “I would change the four outdoor periods depending on the season.”
	ECE training	• “Brainstorm with providers to create sustainable and realistic ideas.”	• “To help with the policy is to teach them [ECEs] how to interact and engage with the children in like a song or a dance.”

*Note.* PA = physical activity; ECE = early childhood educator; SPACE = Supporting Physical Activity in the Childcare Environment; MVPA = moderate-to-vigorous intensity physical activity; PLAY = Childcare PhysicaL ActivitY.

### ECEs’ Perspectives of the Policy: Themes, Context, and Enjoyment

Ten ECEs from the experimental condition agreed to participate in interviews. Thirteen distinct themes were referenced by ECEs representing feasibility (*n* = 4), challenges faced(n = 6), and solutions used (*n* = 3) during policy implementation. Overall, ECEs perceived the policy to be enjoyable and reported that having a set of written statements (e.g., the policy document) to follow acted as a reinforcing factor to highlight the importance of physical activity. Challenges during policy implementation included difficulty with transition periods moving from indoors to outdoors, lack of knowledge/training regarding structured physical activity, and contextual factors, such as inclement weather. ECEs reported that role modeling and teacher-facilitated physical activity were effective solutions for the aforementioned challenges. In addition, having the space to play indoors when inclement weather was present was also frequently noted. ECEs expressed that participating in the intervention made them aware of their unique childcare center environments and their influence on facilitating or hindering children’s activity affordances. Finally, ECEs expressed that following the policy resulted in better sleep among toddlers and preschoolers. See Table 5 for ECEs’ perceptions regarding challenges, solutions, and feasibility of policy implementation and for their opinions regarding policy effectiveness, enjoyment, and suggestions for improvement.

## Discussion

The purpose of this study was to conduct a process evaluation of the Childcare PLAY Policy. Given that the policy was administered by ECEs, the process evaluation was conducted by examining ECEs’ implementation fidelity, and their perspectives of context, feasibility, enjoyment, and effectiveness, and future implementation of the policy. This is the first Canadian study to examine the implementation of a physical activity–focused policy in childcare through an ECE lens. The results suggest that this intervention was well received, and considered feasible by participants, with some suggestions for policy modification.

ECEs are responsible for daily childcare programming, and their personal attitudes and opinions regarding physical activity are shown to influence their daily curriculum and inclusion of physical activity opportunities (Hesketh et al., 2017). The delivery of interventions, such as the Childcare PLAY Policy, is dependent on proper implementation (i.e., high fidelity; Carroll et al., 2007); the limited policy-specific training (i.e., 30 minutes of in-house instruction) ECEs received prior to implementing the Childcare PLAY Policy may have influenced their ability to deliver the intervention as intended. Existing evidence supports the importance of pre-intervention training sessions on the motivation and self-efficacy of those assigned to implement it (Copeland et al., 2012), and although little training was offered in the present study, the high adherence rates to many policy items demonstrate ECEs’ commitment to implementing the proposed policy.

ECEs are responsible for planning daily curriculums for the children in their care (Hesketh et al., 2017), and previous policy interventions have found that adherence rates may vary as a result of daily fluctuations (Lessard et al., 2014). In the present study, policy items (e.g., providing shorter, more frequent outdoor periods) that were influenced by daily fluctuations (e.g., weather, child-to-ECE ratios) had lower adherence rates compared with the implementation of policy items not affected by daily fluctuations (e.g., high adherence to limiting children’s use of screen-based technology due to lack of such devices in childcare centers) that were easier to control by ECEs and thus had higher rates of compliance. As such, adopting multiple policies and practices (i.e., having a physical activity and/or sedentary time policy to follow paired with normal daily programming requirements) is an additional task added to an already substantial agenda of managing young children. It is possible that the more tasks ECEs are asked to complete in children’s daily routines the less likely that there will be high compliance, as task load may become too difficult or overwhelming to manage. Despite this possibility, participating ECEs reported good adherence to many of the Childcare PLAY Policy items. For example, there was 83% full compliance for encouraging children to engage in fundamental movement skills (e.g., running, skipping) and 93% full compliance for ECEs appropriate role modeling of screen-based technology. ECEs found some items found more challenging, as indicated by low compliance (i.e., 12% full compliance for offering shorter, more frequent outdoor sessions).

In some instances, there are mitigating factors for high compliance. For example, high compliance was seen for providing 120 minutes of outdoor time per day, but this is hardly surprising given that Ontario’s Child Care and Early Years Act, 2014, stipulates this requirement for all childcare centers. Ensuring that children receive sufficient outdoor time is an important policy item, but it must be paired with other policy items, such as sufficient time spent in MVPA, to reap its full effectiveness in increasing children’s activity levels. This policy item may be a more important consideration if the Childcare PLAY Policy were to be implemented outside Ontario, in other provinces that do not statutorily require outdoor playtime (e.g., Saskatchewan, Alberta; Vercammen et al., 2020). For other policy items, high compliance is a promising finding. For example, high compliance to policy items concerning screen-based technology is important and should be considered when designing and implementing childcare center policies given that no provincial legislation exists in Ontario regarding screen use, suggesting ECE buy-in (Vanderloo & Tucker, 2018). Further study is warranted, however, as this high level of compliance may be attributed to the lack of screen-based technology in the childcare centers that participated in this study.

The difficulty with integrating shorter, more frequent outdoor periods into weekly routines was reflected by the lowest adherence of all policy items (i.e., 12% compliance). ECEs expressed that the increased number of indoor/outdoor transitions was the challenge. Considering this study was conducted during the fall and winter months in Ontario, implementation may have been affected by unfavorable weather, which is an important factor in the delivery of childcare-based interventions (Copeland et al., 2012; Edwards et al., 2015; Tandon et al., 2017). During the cooler months, children are required to wear more clothing (e.g., snow suits, winter boots) for outdoor periods, and participating ECEs reported that getting the children dressed was time-consuming and inconvenient to perform multiple times per day. However, given that multiple daily outdoor periods have been identified as effective at increasing physical activity among children in childcare (Alhassan et al., 2013; Wolfenden et al., 2016), effort needs to be focused on creating feasible adaptations for year-round application (e.g., provide indoor physical activity sessions instead). Thus, future studies should investigate whether ECEs’ perspectives of providing shorter, more frequent outdoor periods would differ with the policy implemented during the summer months, when there are fewer requirements to ready children.

Given the young age of children in childcare settings and their reliance on ECEs to offer sufficient activity opportunities, the attitudes and perspectives of ECEs are crucial for future policy improvements. During interviews, ECEs commented that factors unique to their childcare environments (i.e., distinctive aspects of their particular workplace) acted as barriers. For example, ECEs emphasized that due to lack of space in their classroom they were fearful to promote movement in the event that children would “bump” or “knock” into things; some ECEs reported that they were thankful for their large outdoor play area, or indoor gym, as a way to overcome small classrooms. These findings are consistent with a recent systematic review that found the presence of outdoor environments and large indoor play spaces to be associated with higher levels of physical activity (Tonge et al., 2016). Similarly, De Decker et al. (2013) identified similar barriers (e.g., space) through focus groups with ECEs. As a result, factors unique to childcare environments (e.g., existence/absence of indoor gyms) should be considered and discussed with childcare staff prior to implementation of interventions to determine potential obstacles at the outset and to identify appropriate solutions.

Finally, during interviews, ECEs reported that the children in their care slept better during daily naptime on days with high adherence to the policy. Improved sleep behaviors are important, as healthy sleep patterns in young children serve an important role in the prevention of obesity (Bathroy & Tomopolous, 2017) and foster improved emotional regulation (Chaput et al., 2017). Furthermore, it has been found that children who engage in high amounts of screen-viewing have been shown to exhibit poorer sleep quality (Brockmann et al., 2016). While increased sleep time may be attributed to the high adherence to the screen-based technology policy item, or limited access to technology, it nevertheless demonstrates the importance and benefits associated with ensuring that children engage in sufficient physical activity and avoid sedentary time. Future studies should focus on how policies may aid in promoting the successful achievement of all 24-hour movement behaviors (sleep, screen time, and physical activity), and seek to identify the ideal frequency and duration of various policy items (e.g., outdoor sessions, teacher-facilitated activity) that are appropriate for children in childcare.

### Strengths and Limitations

The diversity of the tools used to conduct the process evaluation are a strength of this study; however, several limitations must be considered. First, the adherence to the policy components was based on self-reported data and, therefore, may have been influenced by social desirability bias. Second, only one implementation log was provided per classroom. Therefore, it was unclear whether the same ECE was completing the log each day or if there was any variance in how ECEs completed the log (e.g., different levels of agreement). In fact, it is possible that not all ECEs allocated to the experimental condition were following the policy within a classroom. To overcome this, future studies should implement a way of tracking who completes the daily log. Third, the success of policy implementation could have been affected by a variety of factors not explored in this study, including differences in environmental factors such as indoor/outdoor space of childcare centers, effects of weather during policy implementation, perceived importance of physical activity, ECEs’ quality of physical activity–related training, and unreported childcare staff turnover. Fourth, ECEs implementation was difficult to evaluate because the implementation log was designed to assess more items (*n* = 17) than were presented in the original policy document (*n* = 8). A different implementation log would have proved beneficial and will be created for use in future studies. Fifth, it is possible that ECEs who volunteered to participate in interviews were more invested in the PLAY policy compared with ECEs who did not volunteer, and therefore, had greater adherence and/or positive opinions of the policy. Finally, although the sample consisted of randomly selected childcare centers, all centers were drawn from a limited geographic region and all participants were female, which may limit the generalizability of the findings.

### Conclusion

With nearly half of young Canadian children attending some form of childcare (Statistics Canada, 2019), it is imperative that further policy research be conducted to identify how these settings can provide opportunities for children to engage in healthy movement behaviors. The reported rates of adherence to the Childcare PLAY Policy paired with the positive feedback from participating ECEs illustrate the potential value of this policy for supporting appropriate physical activity and reducing sedentary time. It is important that researchers in the field understand the effects of daily fluctuations (e.g., inclement weather) on ECEs’ ability to implement the policy. As such, future directions should consist of policy modification, in collaboration with important childcare stakeholders (e.g., childcare center directors, advisory councils, policy makers) within the context of the feedback received in this pilot study. In addition to making modifications, the Childcare PLAY Policy needs to be tested on a larger scale. Future studies should provide comprehensive resources (e.g., training) to support optimal knowledge for ECEs who are responsible for delivering such interventions. In conclusion, the results from this study are helpful in determining areas for physical activity policy and program improvement and set the stage for a future outcome evaluation.
